# Phenological Stages of the Species *Jacaranda mimosifolia* D. Don. According to the Extended BBCH Scale

**DOI:** 10.3390/biology14111569

**Published:** 2025-11-09

**Authors:** Ignacio Gandía-Ventura, Isabel López-Cortés, Borja Velàzquez-Martí

**Affiliations:** 1 Departamento de Producción Vegetal, Preservation and Improvement of Valencian Agro-Diversity University Research Institute (COMAV), Universitat Politècnica de València, Camino de Vera s/n, 46022 Valencia, Spain; islocor@upv.es; 2 Departamento de Ingeniería Rural y Agroalimentaria, Universitat Politècnica de València, Camino de Vera s/n, 46022 Valencia, Spain; borvemar@dmta.upv.es

**Keywords:** jacaranda, BBCH scale, phenology, development stages, characterization, temperature

## Abstract

**Simple Summary:**

*Jacaranda mimosifolia* D. Don. is a popular ornamental tree widely planted in Mediterranean cities for its attractive foliage and purple flowers. However, its seasonal development has never been formally described under these environmental conditions. In this study, we monitored jacaranda trees throughout the year and identified their main growth phases using the standardized BBCH phenological scale. We also calculated the amount of heat required for the species to progress from winter dormancy to leaf fall, known as growing degree-days (GDD). Our results show that jacaranda completes its annual cycle after accumulating about 3800 GDD, indicating that it grows successfully in Mediterranean climates. This information can help improve management practices in ornamental plantings and support decisions regarding the potential use of jacaranda in experimental or agroforestry cultivation systems.

**Abstract:**

The jacaranda (*Jacaranda mimosifolia* D. Don.) is a widely cultivated ornamental tree species in urban landscapes, but recent research has highlighted its additional ecological and industrial potential. However, no detailed phenological description has been available for this species. The objective of this study was to establish a standardized phenological scale for *Jacaranda mimosifolia* D. Don. based on the BBCH coding system and to determine the thermal requirements (growing degree-days, GDD). Thirty-nine secondary stages were used to describe the life cycle of jacaranda in this BBCH scale, distributed across seven principal growth stages (PGSs). Of these thirty-nine secondary stages, five stages correspond to dormancy and sprouting (PGS-0), six stages correspond to leaf development (PGS-1), three correspond to the emergence of the flowering organ (PGS-5), eight correspond to flowering (PGS-6), ten correspond to fruit formation (PGS-7), three correspond to ripening fruit (PGS-8), and four correspond to the beginning of dormancy (PGS-9). Thermal integral analysis indicated that jacaranda requires approximately 3800 accumulated degree-days (GDD) to progress from dormancy to leaf fall. This phenological framework enhances understanding of the growth cycle of *Jacaranda mimosifolia* D. Don. and provides a useful reference for improving the timing and efficiency of management and phytosanitary treatments in Mediterranean conditions.

## 1. Introduction

*Jacaranda mimosifolia* D. Don. is a tree species native to tropical and subtropical regions whose applications are becoming increasingly diverse worldwide. It is mainly used as an ornamental species, widely planted in urban environments as a decorative tree in streets, parks, and squares, both for its beauty and for the shade it provides. However, it is also applied in the recovery and analysis of bioactive compounds with therapeutic relevance, for instance, in the treatment of gastric ulcers and in the extraction of antioxidant compounds and glycosides. Moreover, its use as a reforestation species in degraded environments is an emerging field of research due to its growth performance and adaptability [1,2,3,4,5,6,7].

*Jacaranda mimosifolia* D. Don. originates from Central America [8,9] and belongs to the family *Bignoniaceae* [10]. It is a botanical family widely used in gardening in Spain (particularly along the Mediterranean coast) and as a timber species internationally. Other members of the Bignoniaceae family are also cultivated in gardens and nurseries, such as *Capsidium valdivianum* B. and *Campsis radicans* L. [11,12,13,14] (Fabris, 1959; 1965; 1979; 1993), while *Handroanthus chrysanthus* J. is frequently grown for timber production [15].

**Figure 1:**
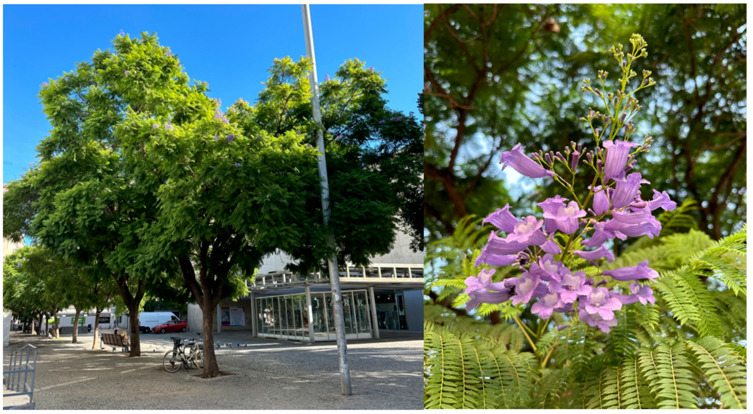
Specimen of *Jacaranda mimosifolia* D. Don. (**left**) and detail of flowering (**right**).

These trees can reach heights up to 12 m, with a crown spread of up to 18 m wide. Their crown is globose, with bipinnate, opposite, large leaves (30–60 cm long) containing numerous small leaflets [16]. The flowers are large, bell-shaped, hermaphrodite, and arranged in terminal panicles (occasionally axillary), measuring 25–30 cm in length [17] (Figure 1). The fruit is a woody, disc-shaped, dehiscent capsule that remains on the tree for long periods, opening to release the seeds [18]. According to López [19], seeds are small, brown, circular (7–9 mm in diameter), and surrounded by a transparent membrane.

To better understand the adaptation of *Jacaranda mimosifolia* D. Don. to new environments, it is necessary to conduct phenological monitoring of the species. Phenology is the science that studies cyclical biological phenomena, such as budburst, flowering, fruit growth, and leaf fall, which are influenced by climatic variables including temperature and photoperiod [20,21,22,23,24,25,26]. Understanding these eco-biological processes and their relationship with climate makes it possible to evaluate the species’ responses to different environments and assess its capacity for adaptation [27,28,29,30], which represents one of the major challenges in current ecological research [31]. Furthermore, accurate knowledge of phenological stages enables more efficient and environmentally sustainable management practices, particularly regarding the optimal timing of phytosanitary treatments in cultivation [32].

For instance, flowering is determined by the temperature in the months preceding floral development [33]. Moreover, species within the same botanical family often share similar flowering schedules, meaning that knowledge of one taxon can provide insights into the phenological behavior of related species under different environmental conditions [34]. The use of thermal time approaches, such as growing degree-days (GDD), has become widely adopted to quantify heat requirements and predict the timing of key phenophases, while different formulations of temperature-sum models can be applied depending on the phenological context [35].

Efforts to classify phenological stages have evolved over time. One of the first systematic approaches was developed by Zadoks et al. [36], who proposed a decimal code assigning two digits to the phenological stages of cereals, homogenized across species. Earlier attempts included the detailed phenological tables by Fleckinger [37,38] for fruit trees and the letter-coded scale (A–I) by Baggiolini [39] for grapevines. Aubert and Lossois [40] later proposed a ten-stage system and established some of the first phenological observation networks.

Building upon Zadok’s work, the Federal Biological Research Centre for Agriculture and Forestry in Germany developed species-specific scales in 1979, in which the first digit represented the primary stage and the second digit the secondary stage [41]. However, this early scale did not provide a universal framework applicable to all plant species [42]. This limitation was later addressed through the development of the Biologische Bundesanstalt, Bundessortenamt, und Chemische Industrie (BBCH) scale [43], which standardized phenological observations using a two-digit decimal code, later expanded to an extended version with three digits to improve stage precision [44,45,46]. Stauss [47] compiled these codes for various species into a unified reference manual for field use.

Since then, the extended BBCH scale has been widely applied to many crops to describe their biological cycle, including *Solanum betaceum* Cav [48], *Mangifera indica* L. [49], *Persea americana* Mill. [50], *Solanum muricatum* Aiton [51], *Olea europaea* L. [52], *Abelmoschus manihot* L. [30], *Junglans regia* L. [28], and *Buddleja saligna* Willd. [29].

Species-specific phenological characterization is essential to understand adaptation and survival in different environments. Therefore, monitoring the phenology of *Jacaranda mimosifolia* D. Don. enables a deeper understanding of its biological cycle and behavior across environmental gradients, providing the necessary information to evaluate its potential for cultivation under Mediterranean conditions. In this context, the present study applies the extended BBCH scale to describe the complete phenological development of *Jacaranda mimosifolia* D. Don. and quantify its thermal requirements through growing degree-days (GDD). We hypothesize that the species follows a consistent BBCH progression governed by temperature accumulation, confirming its phenological compatibility with Mediterranean climates.

## 2. Materials and Methods

In order to know the phenology of *Jacaranda mimosifolia* D. Don., a phenological monitoring of the species was carried out on individuals located in the municipality of Bétera, Valencia, Spain.

### 2.1. Study Site and Plant Material

The study was conducted in Bétera (Valencia, Spain), at 92 a.m.s.l. (Spindle 30N X:717949.708 Y:4385462.519), under Mediterranean climatic conditions [53].

Fifty adult *Jacaranda mimosifolia* D. Don. trees implanted in a unique orchard were monitored. Trees were approximately 15 m in height and 40 cm in trunk diameter, with an estimated age of 20 years. All trees were free from visible biotic or abiotic stress and under standard management.

### 2.2. Field Experiment and Phenological Observations

Phenological monitoring was carried out weekly during the 2022 and 2023 growing seasons. For each tree, the predominant phenological stage was recorded according to the extended BBCH scale, and the most advanced and delayed stages were additionally noted to characterize intra-individual variability. A total of 40 secondary growth stages were defined and allocated to seven principal growth stages. Each phenological stage observed was assigned its description according to the BBCH scale and compared with the classic scale of Aubert and Lossois [40].

Phenological stages were assigned based on morphological criteria following the extended BBCH system. Representative photographs were taken monthly for each principal growth stage identification.

### 2.3. Thermal Integral Calculation

Daily maximum and minimum air temperature data were obtained from the nearest official meteorological station (IVIA Bétera, 2 km from the study site). Growing degree-days were calculated as follows [54,55]:

(1) $$GDD=\max\left(0,\frac{T_{max,d}+T_{min,d}}{2}-T_{0}\right)$$

where $T_{0}=7\;{}^{\circ}C$ was selected as the base temperature [56]. Cumulative GDD was computed from the onset of bud development (BBCH 00) until leaf fall (BBCH 97).

## 3. Results

### 3.1. BBCH Phenological Codification

A total of thirty-nine secondary BBCH stages were recorded and grouped into seven principal growth stages (Table 1). Each stage was defined by clear morphological descriptors, allowing reproducible stage assignment in field observations. The BBCH scale applied here provides a standardized reference framework for future phenological studies and comparative analyses across environments and years.

### 3.2. Visual Characterization of Principal Phenophases

**Figure 2:**
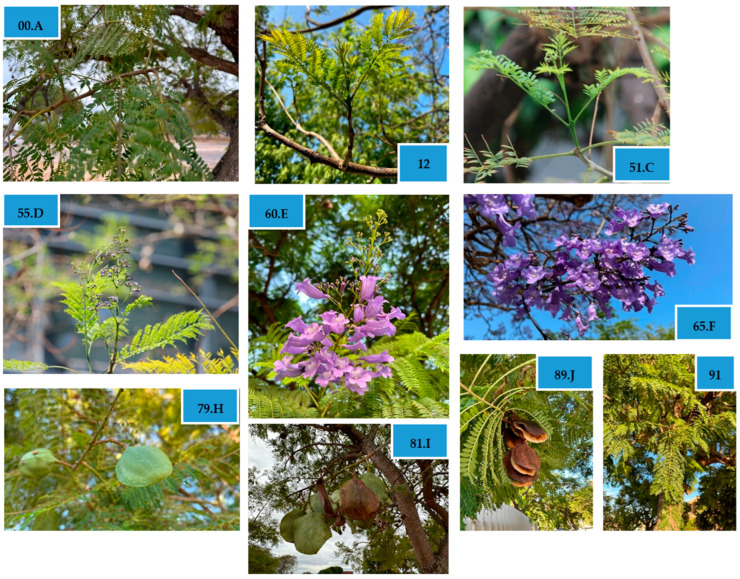
Vegetative growth stages of *Jacaranda mimosifolia* D. Don., according to the BBCH scale and the classic scale of Aubert and Lossois [40]: (**00.A**) winter dormancy or resting period; (**12**) development of the second leaf; (**51.C**) flower organs or flower buds visible; (**55.D**) first individual buds and flower buds (florets) visible (unopened); (**60.E**) first flowers, open; (**65.F**) full flowering: 50% of flowers open; first petals fall off or dry up; (**79.H**) fruits have reached the size appropriate to their species/variety; (**81.I**) beginning of ripening or fruit coloring; (**89.J**) full ripening or harvesting; end of species-typical coloring; (**91**) end of wood or shoot growth (shoots), green foliage.

All principal stages are illustrated in this section (Figure 2):

### 3.3. Principal Growth Stage 0—Germination, Sprouting, Bud Dormancy

PGS 0 marks the transition from winter quiescence to the beginning of metabolic activation in *Jacaranda mimosifolia* D. Don. During this stage, buds remain closed until they show swelling at the apex, indicating the resumption of cell division following the colder period. In both years monitored, this phase consistently occurred from January to February, when no heat accumulation seems to be required (0 GDD). This high stability suggests that jacarandas respond reliably to winter conditions in Mediterranean climates.

### 3.4. Principal Growth Stage 1—Leaf Development

Leaf development (PGS 1) began with the unfolding of the large bipinnate leaves characteristic of jacaranda, progressively increasing canopy area and enabling photosynthetic activation. In 2022, this stage was reached on 6th April, after accumulating 360 GDD, whereas in 2023 it occurred six days later, on the 2nd of April, requiring slightly more heat (382 GDD). Despite this small difference, both years followed a similar development trend, indicating a moderate temperature dependence during early vegetative growth. The initiation of PGS 1 coincides with rising spring temperatures, confirming that the vegetative push of *Jacaranda mimosifolia* D. Don. is highly coordinated with the onset of favorable environmental conditions.

### 3.5. Principal Growth Stage 5—Emergence of the Flowering Organ

PGS 5 corresponds to the appearance of the first inflorescences after leaf development, marking the onset of the reproductive phase. This process was clearly detectable by mid-April in both seasons: 10 May 2022 and 7 May 2023, requiring 648 and 693 GDD, respectively. The nearly identical timing suggests that variation in thermal accumulation earlier in the season does not strongly accelerate or delay the reproductive switch. This stability may indicate a well-established synchronization of reproductive onset with Mediterranean climatic conditions.

### 3.6. Principal Growth Stage 6—Flowering

Flowering (PGS 6) was one of the most visually noticeable phenophases, with abundant bell-shaped bluish-purple flowers dominating the crown. In 2022, full flowering occurred on 5 June 2022, requiring 992 GDD, while in 2023 it occurred on the same day, at 1020 GDD. Importantly, both seasons positioned flowering squarely in mid-June, confirming the suitability of jacaranda for ornamental interest during late spring across Mediterranean urban environments and a critical moment for pest attacks.

A secondary flowering event was observed in a few trees in early September, but it represented only approximately 15–20% of the inflorescence abundance recorded during the main spring flowering peak.

### 3.7. Principal Growth Stage 7—Fruit Formation

Fruit formation began soon after petal senescence, with the development of green woody capsules. This phase was observed on 16 July 2022, requiring 1725 GDD, and on 23 July 2023, requiring 1887 GDD. The later occurrence in 2023 can reflect a slower thermal accumulation in late spring, highlighting a higher sensitivity of reproductive development to climatic variability compared with earlier stages. Nonetheless, the formation of fruits occurred well before the temperature decline of late summer, ensuring successful fruit maturation.

### 3.8. Principal Growth Stage 8—Ripening and Fruit Coloring

During PGS 8, capsules increased in size, hardened, and transitioned to a brown coloration. This phase is key to the dispersal readiness of the species. Fruit ripening occurred in mid-October in 2022 (13 October 2022; 3291 GDD) and approximately two weeks later in 2023 (31 October 2023; 3521 GDD). The greater difference between years in this stage suggests that post-set development is more influenced by heat supply and summer temperature trends. Despite this variability, jacaranda fruit consistently ripens before the onset of autumn low-temperature constraints.

### 3.9. Principal Growth Stage 9—Beginning of Dormancy

The final phase of the annual cycle involves progressive senescence and abscission of leaves, allowing the tree to enter dormancy. PGS 9 occurred in mid-December both years examined: 14 December 2022 and 18 December 2023, requiring 3853 GDD and 3882 GDD, respectively. The convergence of thermal requirements toward the end of the cycle is able to complete this development in Mediterranean environments. The reliable completion of phenology is important for long-term establishment and sustainability in landscapes and orchard settings.

### 3.10. Annual Cumulative Thermal Patterns

**Figure 3:**
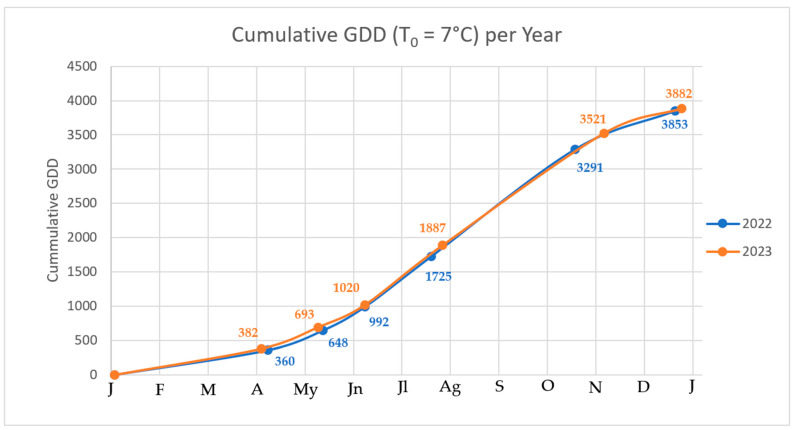
Cumulative GDD (T_0_ = 7 °C) per year of the phenophases of *Jacaranda mimosifolia* D. Don.

The cumulative GDD curves (Figure 3) illustrate a parallel thermal progression between the two monitored years, with slight variations in the rate of accumulation during spring and early summer. The result concorded with the ones obtained for other species [28,29,30].

In 2022, GDD accumulation increased more rapidly during late May and June, while 2023 showed a more regular trajectory extending into July. However, both years converged to 3800 GDD (T_0_ = 7 °C) by mid-December, ensuring full phenological completion.

### 3.11. Statistical Comparison of Thermal Requirements Between Years

To assess interannual consistency in thermal demands, we compared the accumulated GDD required for the onset of each principal BBCH stage in 2022 and 2023 (Table 2). Although GDD values were slightly higher in 2023, thermal variability within each year was low (C% < 8% for all phenophases), indicating stable heat requirements among trees.

## 4. Discussion

The phenological framework developed in this study represents the first standardized description of the development stages of *Jacaranda mimosifolia* D. Don. under Mediterranean conditions, integrating the extended BBCH coding system with thermal accumulation models. The close agreement observed between the two monitored years demonstrates a highly stable phenological pattern despite typical seasonal variability in temperature. Although some statistically significant differences in heat requirements were detected between 2022 and 2023 (*p* < 0.05), the magnitude of interannual variation remained small (CV% < 8% in all stages), indicating that the differences are not biologically meaningful. Similar interannual stability has been reported for other woody ornamentals and fruit trees evaluated using BBCH scales, such as *Junglans regia* L. [28], *Buddleja saligna* Willd. [29], and *Abelmoschus Manihot* L. [30].

The cumulative thermal requirement of approximately 3800 GDD (T_0_ = 7 °C) to complete the seasonal cycle confirms that *Jacaranda mimosifolia* D. Don. is well suited to warm temperatures and Mediterranean conditions. These results suit the ones obtained for *Corylus avellana* L. by Taghavi et al. [57], who determined that hazelnuts can adapt for climates similar to their own experimental zone.

Urban environments and orchards are experiencing increasingly variable climate patterns, and rising temperatures could modify plant development and ornamental quality in the coming decades. However, if spring warming continues to advance, the onset of key phenophases such as flowering could shift, potentially altering interactions with pollinators, modifying flowering displays and affecting fruit maturation [58].

The integration of BBCH phenological monitoring with thermal accumulation models has direct applications for sustainable orchard management. In ornamental trees such as *Jacaranda mimosifolia* D. Don., correctly anticipating leaf expansion, flowering, or fruit set can optimize cultural practices and reduce maintenance costs. The precise identification of phenophases enables improved scheduling of pruning, fertilization, and irrigation, particularly in urban landscapes where management resources are limited. In addition, knowing the optimal phenophase windows may enhance the efficacy of phytosanitary interventions while minimizing repeated chemical applications, contributing to more environmentally responsible practices [32,58,59].

## 5. Conclusions

This study provides the first standardized phenological description of *Jacaranda mimosifolia* D. Don. under Mediterranean conditions, integrating the extended BBCH scale with the thermal accumulation model.

Thirty-nine second growth stages were consistently identified along seven principal growth stages across two consecutive years, and their onset was associated with reproducible GDD thresholds (CV% < 8). The cumulative thermal requirements of approximately 3.800 GDD (T_0_ = 7 °C) suggest that jacaranda is well adapted to warm temperate climates.

The BBCH-GDD framework developed here offers a robust baseline for future ecological studies, climate-impact assessments, and comparative phenology of ornamental woody species. Continued monitoring across broader climatic gradients and longer time series will further refine predictive capacity and support informed use of *Jacaranda mimosifolia* D. Don. in climate-resilient orchards and landscape planning.

This research team intends to analyze in the future the phenology of *Jacaranda mimosifolia* D. Don. in other ecosystems, in comparison with the phenological patterns described in this study.

## Figures and Tables

**Table 1:** Description of phenological growth stages of *Jacaranda mimosifolia* D. Don. according to extended BBCH scale (letters after Albert and Loisee).

PSG	BBCH Code	Description	Period
0 Germination, sprouting, and development	00 (A)	Winter dormancy or resting period	Jan–Feb.
	01	Swelling of the yolk begins	
	03	End of the yolk begins	
	07	The yolk begins to open or sprout	
	09	The bud shows green shots	Early April
1 Leaf development	10	First leaves separate from the shoot	Early April
	11	Development of the first leaf	
	12	Development of the second leaf	Mid-April
	13	Development of the third leaf	
	1…	Continuation of stages until…	
	19	Development of nine leaves or more	
5 Emergence of the flowering organ	51 (C)	Flower organs or flower buds visible	Mid-May
	55 (D)	First individual buds and buds (florets) visible (unopened)	
	59	First petals (flower leaves) visible	
6 Flowering	60 (E)	First flowers, open	Early June
	61	Beginning of flowering: 10% of flowers open	
	62	20% of open flowers	
	63	30% of open flowers	
	64	40% of open flowers	
	65 (F)	Full flowering: 50% of flowers open	Mid-June
	67	Flowering coming to an end: most petals fallen or dry	
	69	End of flowering: fruit set visible	
7 Fruit formation	70	First visible fruits	Mid-July
	71	Fruits reach 10% of their final size	
	72	Fruits reach 20% of their final size	
	73	Fruits reach 30% of their final size	
	74	Fruits reach 40% of their final size	
	75	Fruits reach 50% of their final size	
	76	Fruits reach 60% of their final size	
	77	Fruits reach 70% of their final size	
	78	Fruits reach 80% of their final size	
	79 (H)	The fruits have reached the size appropriate to their species/variety	Mid-September
8 Ripening and fruit coloring	81 (I)	Beginning of ripening or fruit coloring	Mid-October
	85	Continuation or fruit coloring according to species/variety	Early Nov.
	89 (J)	Full or harvest maturity	Late Nov.
9 Beginning of dormancy	91	End of wood or shoot growth, but foliage remains green	Early Dec.
	93	Beginning of leaf discoloration or leaf drop	
	95	50% of leaves discolored or fallen off	
	97	End of leaf fall. The plant is in winter dormancy or vegetative rest	Mid Dec.

**Table 2:** Descriptive statistics comparison of accumulated growing degree-days (GDD, T_0_ = 7 °C) required to reach the onset of principal BBCH stages in *Jacaranda mimosifolia* D. Don. during 2022 and 2023.

PGS	Mean GDD 2022	SD	CV%	Mean GDD 2023	SD	CV%	*p*-Value (Wilcoxon)
0	0.00	-	-	0.00	-	-	-
1	359.52	25.44	7.08	381.70	24.97	6.54	0.00063
5	647.12	28.58	4.42	693.40	28.86	4.16	2.3 × 10^−9^
6	991.48	22.74	2.29	1019.48	22.35	2.19	2.8 × 10^−7^
7	1725.40	41.69	2.42	1887.22	41.67	2.21	3.5 × 10^−15^
8	3290.60	70.30	2.14	3521.28	69.76	1.98	1.8 × 10^−15^
9	3852.36	65.55	1.70	3882.10	64.14	1.65	0.020

## Data Availability

The data presented in this study are available on request from the corresponding author.
